# Supplementary material for: Neuron-reactive KIR+CD8+ T cells display an encephalitogenic transcriptional program in autoimmune encephalitis
Source: Nat Commun. 2025 Sep 29;16:8568. doi: 10.1038/s41467-025-63573-1 (PMC12479921; doi:10.1038/s41467-025-63573-1)
Supplement: Supplementary file 1 — Supplementary Information [file 41467_2025_63573_MOESM1_ESM.pdf]

Supplementary information

Perriot, Jones et al.

Corresponding author:

Prof. Renaud Du Pasquier

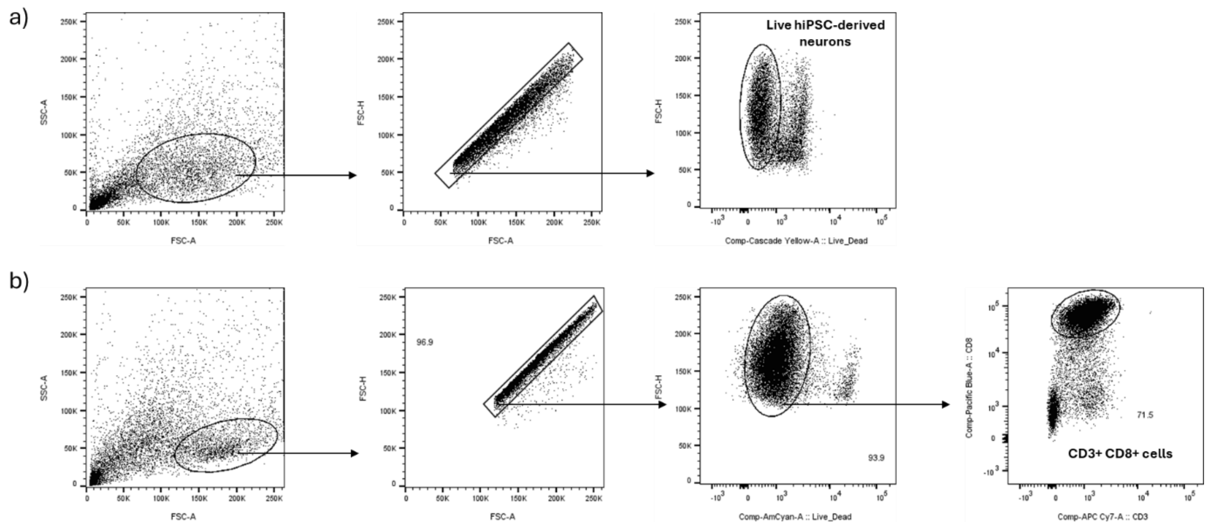

Supplementary Figure 1. Gating strategies for fluorescence-associated cell sorting (FACS).

a) Gating strategy to sort live human-induced pluripotent stem cell-derived neurons presented in Fig. 1b. b) Gating strategy to sort $CD3^{+}CD8^{+}$ T cells from neuron-T cell overnight cultures presented in Fig. 1c.

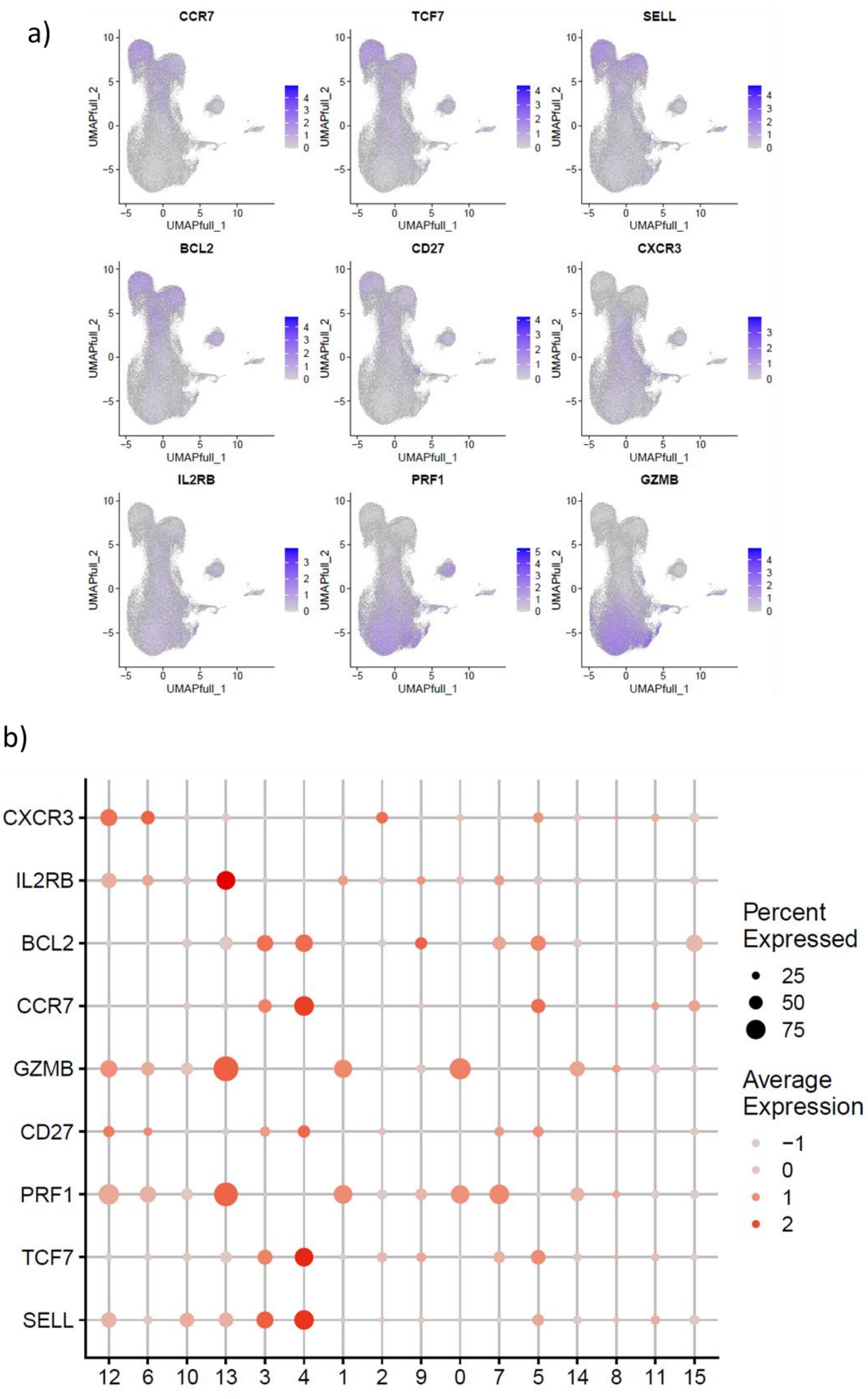

Supplementary Figure 2: Phenotypical assessment of all individual clusters

a) UMAPs displaying expression levels of conventional markers used to classify *ex vivo* naive (*CCR7, SELL, BCL2),* activated (*IL2RB, CXCR3*), cytotoxic (*PRF1, GZMB*) or memory (*CCR7, CD27, TCF7*) CD8^{+} T cells. CD8^{+} T cells presenting with high expression of each respective gene are represented in blue. b) Dot plot displaying the average expression of gene presented in a) across all clusters. Strongly upregulated genes are displayed in intense red with downregulated genes in lighter shades. Size of the dot represents the percentage of cells from each cluster expressing each gene

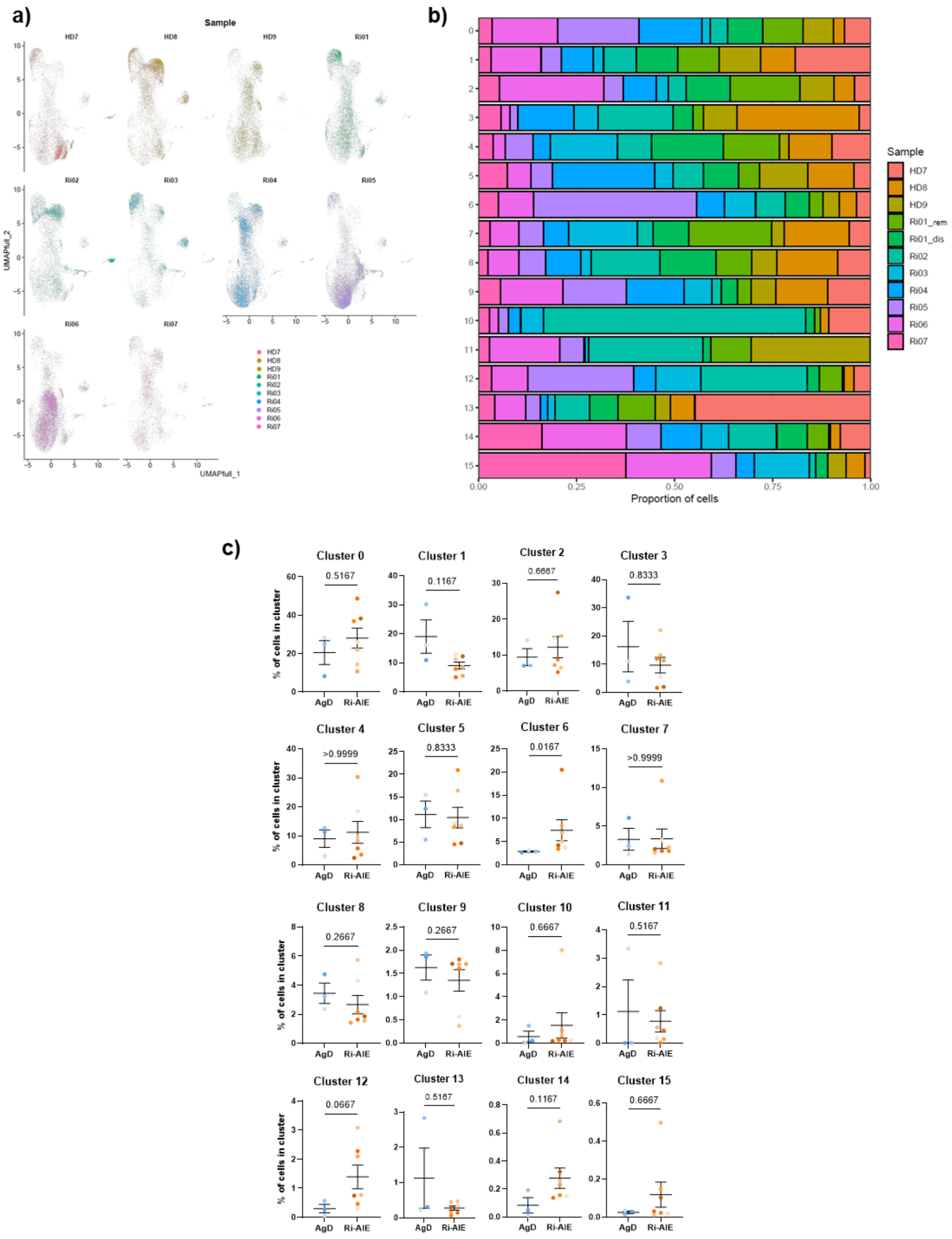

Supplementary Figure 3. Cluster repartition of *ex vivo* CD8$^+$ T cells across all donors assessed by single-cell RNA sequencing

a) UMAP displaying CD8$^+$ T cell repartition across all eleven samples (3 AgD, 7 Ri-AIE and 1 Ri-AIE in remission). Each sample (i.e. donor) is displayed in a different color. b) Bar plots displaying the repartition of each sample across each cluster. Each sample is represented in a different color with bar size representing the proportion that this sample represents relative to other samples. The numbers on the y axis correspond to cluster numbers c) Scatter plot comparing cluster repartition of *ex vivo* CD8$^+$ T cells from AgD (blue) and seven Ri-AIE patients (orange). Horizontal bar represents the overall mean +/- SEM. Statistical significance was assessed using unpaired non-parametric two-sided Mann-Whitney tests (n=3 AgD, 7 Ri-AIE).

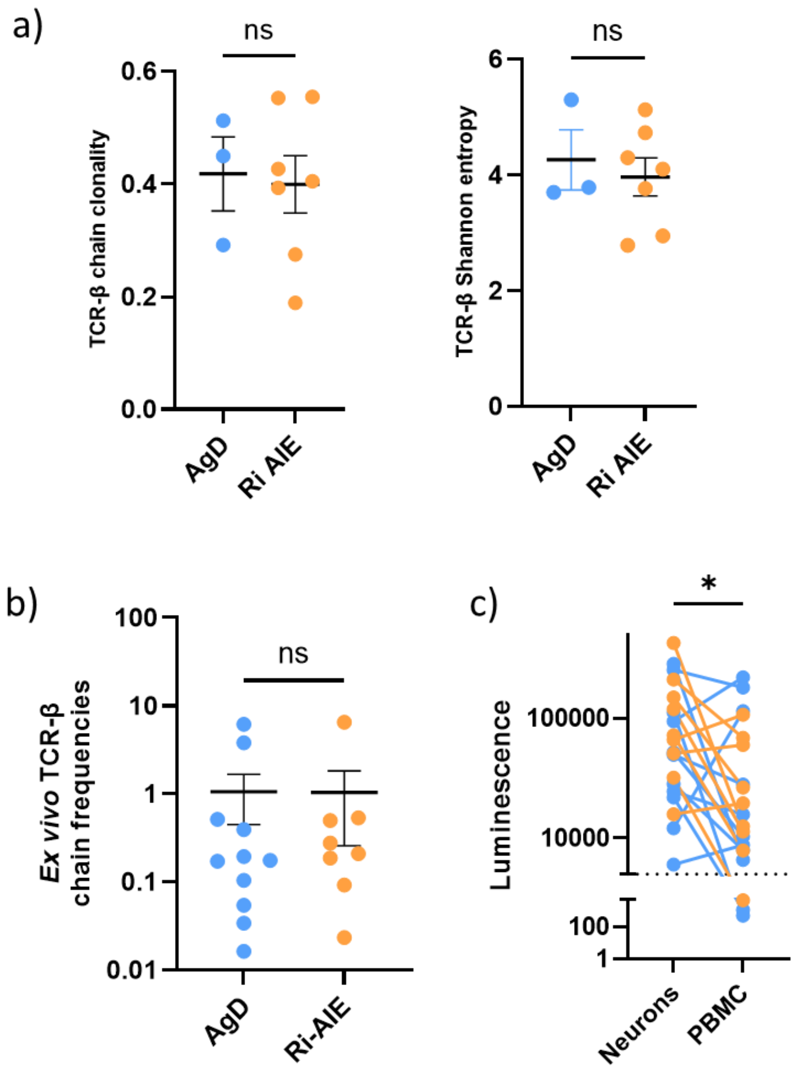

Supplementary Figure 4. Neuron -reactive CD8+ T cells from AgDs and Ri-AIE patients are present at similar frequencies and cross-react with PBMCs

a) Scatter plot comparing TCR-β chain clonality and Shannon entropy of all CD8+ T cell clones identified within cluster 1 between AgD (blue) and Ri-AIE (orange). Horizontal bar represents the overall mean +/- SEM (two-sided Mann-Whitney tests, n=3 AgD, 7 Ri-AIE). b) Scatter plot highlighting *ex vivo* TCR-β chain frequencies of neuron-reactive CD8+ T cell clonotypes found in AgD (blue) and Ri-AIE (orange) as measured by bulk TCR-β chain repertoire sequencing. Horizontal bar represents the overall mean +/- SEM (two-sided Mann-Whitney tests, n=11 AgD, 8 Ri-AIE). c) Luminescence values of NFAT-luciferase Jurkat cells transfected with TCR identified as neuron-reactive in Figure. 4e, from AgD (blue) or RI-AIE (orange) after overnight culture with HLA-enhanced neurons (left) or PBMCs (matched for HLA haplotype). For both neurons and PBMC, a ratio of 1:1 was used between antigen-presenting cell (neuron/PBMC) and effector cell (Jurkat). Horizontal dotted line represents positivity threshold established at a luminescence value of 5’000 (two-sided Wilcoxon test , p value = 0.026, n=20 TCRs in each group).

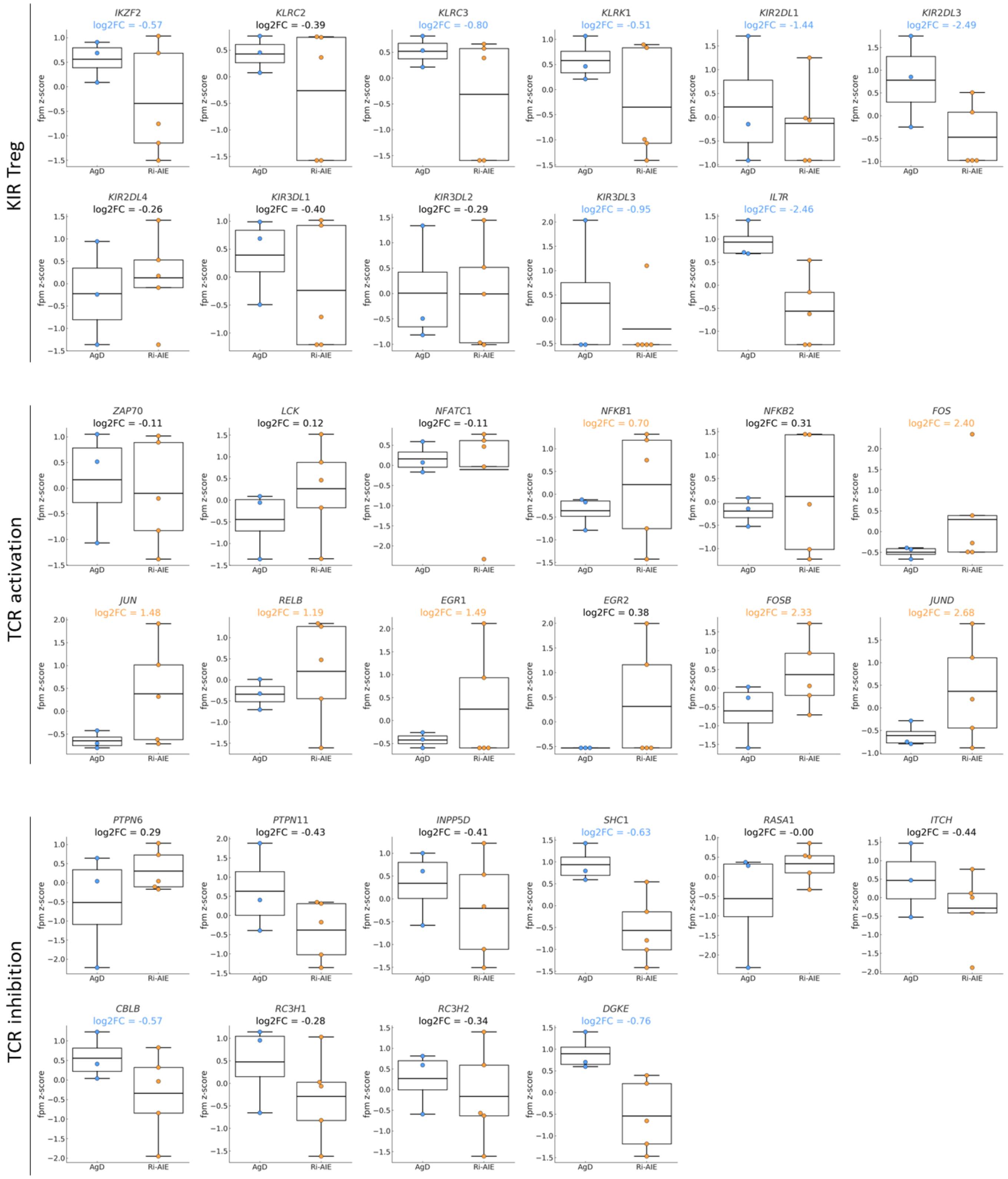

Supplementary Figure 5. Expression of genes associated with KIR Treg, TCR activation and TCR inhibition per donor (pseudobulk aggregation).

Each graph represents the expression of a gene calculated as a z-score of the fragment per million (fpm, counts corrected for differences in fragment number between donors) based on the data aggregated per donor (pseudobulk) including all cells reacting against neurons from cluster 1.

Each dot represents one donor (AgD, left column; Ri-AIE right column). The bar represents the mean expression per group with the error bars the minimum and maximum. Log2FC were calculated using DESeq2 for the comparison AgD vs Ri-AIE. The log2Fc are written in orange when log2FC Ri-AIE vs AgD > 0.5 and in blue when log2FC Ri-AIE vs AgD < -0.5. n=3 AgD, 5 Ri-AIE.

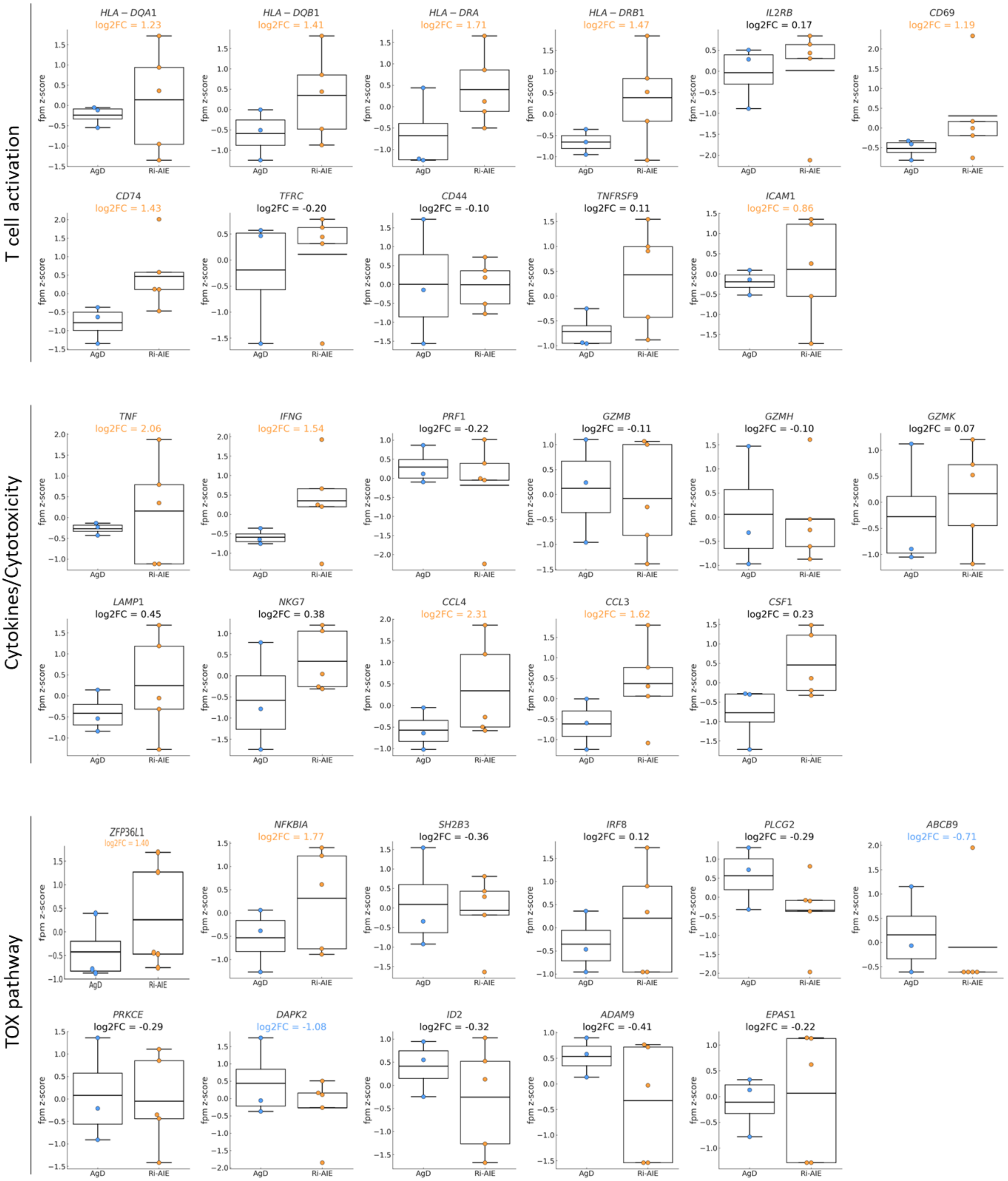

Supplementary Figure 6. Expression of genes associated with T cell activation, cytokine production/cytotoxicity and TOX pathway per donor (pseudobulk aggregation).

Each graph represents the expression of a gene calculated as a z-score of the fragment per million (fpm, counts corrected for differences in fragment number between donors) based on the data

aggregated per donor (pseudobulk) including all cells reacting against neurons from cluster 1. Each dot represents one donor (AgD, left column; Ri-AIE right column). The bar represents the mean expression per group with the error bars the minimum and maximum. Log2FC were calculated using DESeq2 for the comparison AgD vs Ri-AIE. The log2Fc are written in orange when log2FC Ri-AIE vs AgD > 0.5 and in blue when log2FC Ri-AIE vs AgD < -0.5. n=3 AgD, 5 Ri-AIE.

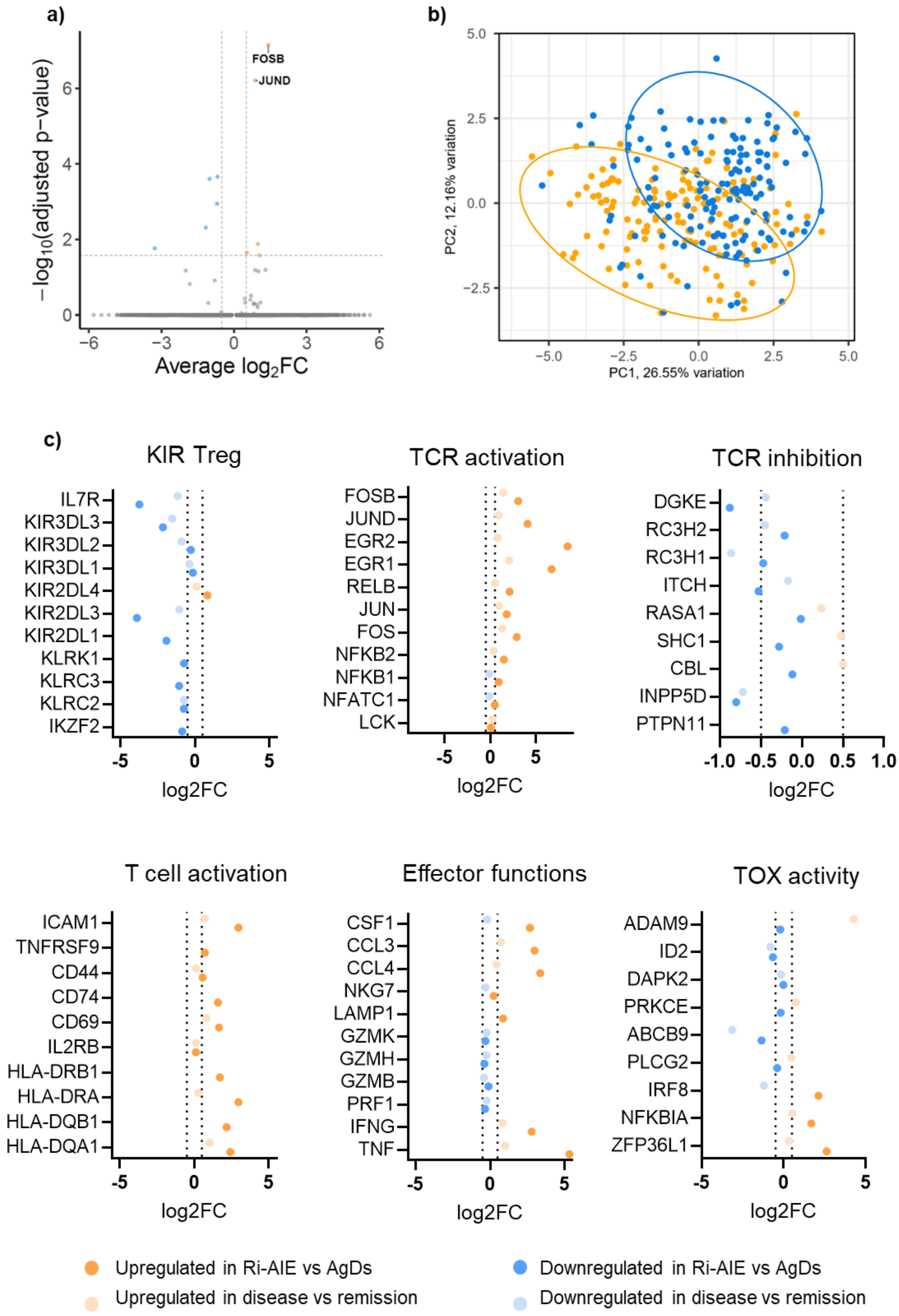

Supplementary Figure 7. Neuron-reactive KIR⁺CD8⁺ T cells in disease vs remission display gene expression trends mimicking the Ri-AIE vs AgD signature.

a) Volcano plot highlighting all significantly upregulated (orange) and downregulated genes (blue) between neuron-reactive KIR⁺CD8⁺ T cell clonotypes from Ri01 patient at the peak of disease vs at remission. Vertical ticked lines represent the fold-change threshold set at an average log_2FC(0.5) for upregulated or log_2FC(-0.5) for downregulated genes. Horizontal ticked line

represents the adjusted p-value threshold established at 0.05. Differentially expressed genes from Figure 5c-h are annotated on the plot. b) Principal component analysis segregating the neuron-reactive KIR^+CD8^+ T cell clonotypes from Ri01 at disease (orange) or remission (blue). PCA was performed according to their expression of the top15 genes dysregulated in Ri-AIE vs AgDs plotted in the heatmaps in figure 5c-h (*JUND, CD74, FOSB, HLA-DRA, ZFP36L1, KIR2DL3, HLA-DRB1, NFKBIA, TNF, CCL4, FOS, EGR2, IL7R, JUN, RELB*). c) Dot plots displaying the log_2FC gene expression between Ri-AIE vs AgDs (upregulated: orange, downregulated: blue) and disease peak vs remission (upregulated: light orange, downregulated: light blue). The genes are selected based on the ones displayed in Figure 5c-h). Vertical ticked lines represent the fold-change threshold set at an average $log_2FC(0.5)$ for upregulated or $log_2FC(-0.5)$ for downregulated genes.

EBV CD8+ T cell-restricted peptide pool					
#	Protein	Peptide location	Amino acid sequence	Presented on HLA[1]	Matched with HD
1	EBNA1	407-417	**HPVGEADYFEY**	B*35:01/08	--
2	EBNA3A	158-166	**QAKWRLQTL**	A*02:01/B*08:01	HD1, HD2, HD3
3	EBNA3A	325-333	**FLRGRAYGL**	B*08:01	HD2
4	EBNA3A	379-387	**RPPIFIRRL**	B*07:02	HD2, HD3
5	EBNA3A	458-466	**YPLHEQHGM**	B*35:01	--
6	EBNA3A	596-604	**SVRDRLARL**	A*02:01	HD1, HD3
7	EBNA3A	603-611	**RLRAEAQVK**	A*03:01	HD2
8	EBNA3B	416-424	**IVTDFSVIK**	A*11:01	--
9	EBNA3C	258-266	**RRIYDLIEL**	B*27:02/05	--
10	EBNA3C	281-290	**EENLLDFVRF**	B*44:05	--
11	EBNA3C	284-293	**LLDFVRFMGV**	A*02:01	HD1, HD3
12	EBNA3C	881-889	**QPRAPIRPI**	B*07:02	HD2, HD3
13	LMP1	125-133	**YLLEMLWRL**	A*02:01	HD1, HD3
14	LMP2	200-208	**IEDPPFNSL**	B*40:01	--
15	LMP2	329-337	**LLWTLVVLL**	A*02:01	HD1, HD3
16	LMP2	340-349	**SSCSSCPLSK**	A*11:01	--
17	LMP2	356-364	**FLYALALLL**	A*02:01	HD1, HD3
18	LMP2	419-427	**TYGPVFMCL**	A*24:02	HD1, HD3
19	LMP2	419-427	**TYGPVFMSL**	A*24:02	HD1, HD3
20	LMP2	426-434	**CLGGLLTMV**	A*02:01	HD1, HD3
21	BMLF1	259-267	**GLCTLVAML**	A*02:01	HD1, HD3
22	BMLF1	397-405	**DEVEFLGHY**	B*18:01	--
23	BRLF1	29-37	**DYCNVLNKEF**	A*24:02	HD1, HD3
24	BRLF1	134-143	**ATIGTAMYK**	A*11:01	--
25	BRLF1	148-156	**RVRAYTYSK**	A*03:01	HD2
26	BZLF1	54-64	**EPLPQGQLTAY**	B*35:01	--
27	BZLF1	190-197	**RAKFKQLL**	B*08:01	HD2
28	BALF4	276-284	**FLDKGTYTL**	A*02:01	HD1, HD3
29	BMRF1	259-267	**YRSGIIAVV**	B*39:06	--

[1]all HLA presentations of distinct epitopes were established based on the immune epitope database (IEDB) and JPT Peptide Technologies indications

EBV: Epstein-Barr virus, HLA: human leukocyte antigen, HD: healthy donor

Supplementary Table 1: Peptide sequences of EBV viral peptide pools used to stimulate ex vivo CD8+ T cells

CMV CD8+ T cell-restricted peptide pool					
#	Protein	Peptide location	Amino acid sequence	Presented on HLA[2]	Matched with HD
1	IE1	88-96	QIKVRVDMV	B*08:01	HD2
2	IE1	81-89	VLAELVKQI	A*02:01	HD1, HD3
3	IE1	184-192	KLGGALQAK	A*03:01	HD2
4	IE1	199-207	ELKRKMIYM	B*08:01	HD2
5	IE1	279-287	CVETMCNEY	A*01:01	HD2
6	IE1	297-304	TMYGGISLL	A*02:01	HD1, HD3
7	IE1	309-317	CRVLCCYVL	C*07:02	HD2, HD3
8	IE1	316-324	VLEETSVML	A*02:01	HD1, HD3
9	IE1	354-363	YILGADPLRV	B*13:02	--
10	IE1	379-387	DEEDAIAAY	B*18:01	--
11	pp50	245-253	VTEHDTLLY	A*01:01	HD2
12	pp65	7-15	RCPEMISVL	C*01:02	--
13	pp65	14-22	VLGPISGHV	A*02:01	HD1, HD3
14	pp65	16-24	GPISGHVLK	A*11:01	--
15	pp65	113-121	VYALPLKML	A*24:02	HD1, HD3
16	pp65	120-128	MLNIPSINV	A*02:01	HD1, HD3
17	pp65	123-131	IPSINVHHY	B*35:01	--
18	pp65	155-163	QMWQARLTV	B*52:01	--
19	pp65	186-196	FVFPTKDVALR	A*68:01	--
20	pp65	188-195	FPTKDVAL	B*35:08	--
21	pp65	198-206	VVCAHELVC	C*08:01/C*15:02	--
22	pp65	199-207	ELRRKMMYM	B*08:01	HD2
23	pp65	215-223	KMQVIGDQY	B*15:01	HD1
24	pp65	232-240	CEDVPSGKL	B*40:01	--
25	pp65	265-274	RPHERNGFTV	B*07:02	HD2, HD3
26	pp65	267-275	HERNGFTVL	B*40:01	--
27	pp65	294-302	VAFTSHEHF	C*12:02	--
28	pp65	341-349	QYDPVAALF	A*01:01	HD2
29	pp65	363-373	YSEHPTFTSQY	A*01:01	HD2
30	pp65	364-373	SEHPTFTSQY	A*01:01	HD2
31	pp65	367-379	PTFTSQYRIQGKL	B*38:01/02	--
32	pp65	369-379	FTSQYRIQGKL	A*24:02	HD1, HD3
33	pp65	415-429	RKTPRVTGGGAMAGA	B*07	HD2, HD3
34	pp65	417-426	TPRVTGGGAM	B*07:02	HD2, HD3
35	pp65	495-503	NLVPMVATV	A*02:01	HD1, HD3
36	pp65	501-509	ATVQGQNLK	A*11:01	--
37	pp65	511-525	QEFFWDANDIYRIFA	B*08/B*44	HD2
38	pp65	515-523	DANDIYRIF	B*08:01/B*35:01	HD2
39	pp65	522-530	RIFAELEGV	A*02:07	--
40	pp65	525-534	AELEGVWQPA	B*40:06	--
41	pp65	545-552	DALPGPCI	B*51:01	HD1
42	pp150	945-955	TTVYPPSSTAK	A*03:01	HD2
43	IE1 (MCMV)	168-176	YPHFMPTNL	--	--
44	m45 (MCMV)	985-993	HGIRNASFI	--	--
45	m164 (MCMV)	257-265	AGPPRYSRI	--	--

Supplementary Table 2: Peptide sequences of CMV viral peptide pools used to stimulate ex vivo CD8+ T cells

	TRAV	CDR3_amino_acid_sequence	TRAJ	TRBV	CDR3_amino_acid_sequence	TRBJ	Ex vivo TCR-β chain frequency (%)	Day 14 TCR-β chain frequency (%)
Neuron-reactive clonotypes	hTRAV22	CAVDGDDKIIFG	hTRAJ30	hTRBV02	CASSLGQGRAWETQYFG	hTRBJ02-5	Undetectable	1.815
	hTRAV13-1	CAAIKSTGNQFYFG	hTRAJ49	hTRBV19	CASSMNSGRGGLLNEQFFG	hTRBJ02-1	Undetectable	0.136
	hTRAV29	CAASDSGGSNYKLTFG	hTRAJ53	hTRBV14	CASSRGTSFYNEQFFG	hTRBJ02-1	Undetectable	0.166
	hTRAV26-2	CILRDGYNNDMRFG	hTRAJ43	hTRBV14	CASSQSGGNGYTFG	hTRBJ01-2	Undetectable	0.140
	hTRAV21	CALGAYYGGSQGNLIFG	hTRAJ42	hTRBV10-3	CASVKSGTENYEQYFG	hTRBJ02-7	Undetectable	0.336
	hTRAV35	CAGLYQAGTALIFG	hTRAJ15	hTRBV07-9	CASSLGGAIMNTEAFFG	hTRBJ01-1	Undetectable	0.116

Non-reactive clonotypes	hTRAV35	CAGRVTGGGNKLTFG	hTRAJ10	hTRBV20	CSARNGNQPQHFG	hTRBJ01-5	0.316	0.345
	hTRAV14	CAMRGAGGYQKVTFG	hTRAJ13	hTRBV06-2	CASSLLGQPWYEQYFG	hTRBJ02-7	0.040	0.057
	hTRAV19	CALSESNDYKLSFG	hTRAJ20	hTRBV27	CASSLSSVPSYEQYFG	hTRBJ02-7	0.016	0.020
	hTRAV12-1	CVVNRSLSGGADGLTFG	hTRAJ45	hTRBV27	CASSLSSVPSYEQYFG	hTRBJ02-7	0.016	0.020
	hTRAV12-4	CAIEDSWGKLQFG	hTRAJ24	hTRBV05-5	CASSLRVNTEAFFG	hTRBJ01-1	0.633	0.968
	hTRAV26-1	CIVRDISSGSARQLTFG	hTRAJ22	hTRBV05-1	CASSWGGRGMNTEAFFG	hTRBJ01-1	1.336	0.797

Supplementary Table 3. List of tested clones for HD4 and their respective frequencies ex vivo and after co-culture

Donor	Sex	Age at PBMC sampling[2] *(age range in years)*	Ethnicity	Condition	Months between disease initiation and sampling[3]	Immunomodulatory treatment at sampling	Reference center for samples
HD1[1]	F	28-32	White	Healthy	--	--	Lausanne, CHUV
HD2[1]	M	48-52	White	Healthy	--	--	Lausanne, CHUV
HD3[1]	F	48-52	White	Healthy	--	--	Lausanne, CHUV
HD4[1]	F	33-37	White	Healthy	--	--	Lausanne, CHUV
HD5[1]	M	33-37	White	Healthy	--	--	Lausanne, CHUV
HD6[1]	F	33-37	White	Healthy	--	--	Lausanne, CHUV
	**F/M n: 4/2**	**Median age ± SD: 36 ± 7.92**					
AgD1	F	68-72	White	Cervical spinal stenosis	12	--	Lausanne, CHUV
AgD2	F	73-77	White	Stroke	1	--	Lausanne, CHUV
AgD3	F	63-67	White	Post-traumatic cervical myelopathy	18	--	Lausanne, CHUV
	**F/M n: 3/0**	**Median age ± SD: 72 ± 5.13**			**Median months ± SD: 12 ± 8.62**		
Ri01[1]	F	68-72	White	RI-AIE	1	None	Lausanne, CHUV
Ri02	F	68-72	White	Ri-AIE	3	None	Lausanne, CHUV
Ri03	F	73-77	White	RI-AIE	4	None	Münster, UKM
Ri04	F	73-77	White	Ri-AIE	10	None	Münster, UKM
Ri05	F	58-62	White	RI-AIE	5	None	Lyon, CHU
Ri06	F	73-77	White	Ri-AIE	3	None	Lyon, CHU
Ri07	F	63-67	White	RI-AIE	14	n/a	Lyon, CHU
	**F/M n: 7/0**	**Median age ± SD: 69 ± 5.94**			**Median months ± SD: 4 ± 4.61**		

[1]All hiPSC cell lines used for this study are listed here according to the hPSCreg nomenclature (LNISi001-B, LNISi002-B,LNISi003-A, LNISi004-B, LNISi005-A, LNISi006-A, LNISi009-B, LNISi010-A, LNISi011-A, LNISi012-A, LNISi016-B, LNISi018-A, LNISi019-A)

[2.] For HD1-6 and for Ri01, hiPSC were reprogrammed at the time of PBMC sampling. For AgD1-3 and for Ri02-07, no hiPSC were reprogrammed

[3] All Ri AIE patients presented with disease-related symptoms at time of blood draw

HiPSC: human-induced pluripotent stem cells, PBMC: peripheral blood mononuclear cells, HLA: human leukocyte antigen, HD: healthy donor, F: female, M: male, F/M: female-to-male ratio, SD: standard deviation, AgD: aged donor, AIE: autoimmune encephalitis, n/a: data not available

Supplementary Table 4: Healthy donor (HD), aged donor (AgD) and Ri-AIE patient demographics

	Ri01	Ri02	Ri03	Ri04	Ri05	Ri06	Ri07
**Sex**	F	F	F	F	F	F	F
**Age range (in years)**	68-72	68-72	73-77	73-77	58-62	73-77	63-67
**Clinical features**	Productive aphasia<br>Focal and generalized seizures	Aphasia<br>Generalized seizures<br>Behavioral disorders	Gait ataxia<br>Diplopia<br>Gaze-evoked nystagmus	Extrapyramidal symptoms	Ataxia<br>Diplopia<br>Gaze-evoked nystagmus<br>Oculomotor disturbances<br>Dyspnea	Bladder dysfunction<br>Ataxia<br>Inferior limb weakness and spasticity	Ataxia<br>Spasticity
**CSF**	WBC: <1 cells/µl<br>IS: no<br>Anti-Ri Abs in serum (1:1000) and CSF (1:32)<br>CSF/serum albumin ratio: 26	WBC: 6 cells/µl<br>IS: yes<br>Anti-Ri Abs in serum and CSF (titers n/a)<br>CSF/serum albumin ratio: 9.5	WBC: 12 cells/µl<br>IS: yes<br>Anti-Ri Abs in serum (1:10000) and CSF (1:320)<br>CSF/serum albumin ratio: 14.1	WBC: 3 cells/µl<br>IS: yes<br>Anti-Ri Abs in serum+ (titer: n/a) and CSF (1:500)<br>CSF/serum albumin ratio: 6.8	WBC: 88 cells/µl<br>IS: yes<br>Anti-Ri Abs+ in serum and CSF (titers n/a)<br>CSF/serum albumin ratio: n/a	WBC: 114 cells/µl<br>IS: yes<br>Anti-Ri Abs+ in serum and CSF (titers n/a)<br>CSF/serum albumin ratio: 6.2	WBC: 37 cells/µl<br>IS: n/a<br>Anti-Ri Abs+ in serum and CSF (titers n/a)<br>CSF/serum albumin ratio: n/a
**MRI**	Normal	T2-weighted bilateral temporal hyperintensities	Global atrophy, microangiopathic lesions	T2-weighted bilateral hyperintensities in putamen and caudate nucleus	Diffuse T2-weighted bilateral white matter hyperintensities	Microangiopathic lesions	Microangiopathic lesions
**Underyling cause**	Unknown (no neoplasia)	Small-cell lung carcinoma	Likely ovarian carcinoma (malignant lymph node infiltrate)	Cancer of unknown primary site (squamous cell carcinoma lymph node metastasis)	Breast cancer	Breast cancer	Breast cancer
**Treatment[1]**	Ri-dis: Methyprednisolone IV (acute)<br>Ri-rem: Mycophenolate mofetil + degressive prednisone	Anti-seizure drugs (clonazepam, pheyntoin, levetiracetam and topiramate) + high-dose corticosteroids	Unknown immune modifying therapy<br>Retriperitoneal lymphadenectomy	Plasma exchanges (5 cycles)<br>Corticosteroid pulse therapy (twice)<br>Cyclophosphamide with relay to rituximab	IV Igs<br>Rituximab	Corticosteroids<br>IV Igs<br>Cyclophosphamide	Corticosteroids
**Outcome**	Favorable	Deceased	n/a	Persistent symptoms	Persistent symptoms	Progression of symptoms	Deceased

[1] These treatments were introduced after blood sampling

AIE: autoimmune encephalitis; Abs: antibodies; CSF: cerebrospinal fluid; IS: intrathecal synthesis of immunoglobulins; IV Ig: intra-venous immunoglobulins; WBC: white blood cells; n/a: not available

Supplementary Table 5: Ri-AIE patient clinical description

	HLA class I typing	Matched HLAs[1] (n/6)	Clonotypes screened per donor[2]
AgD1	**A*02:01, A*24:02, B*44:02, B*51:01,** C*05:01, **C*15:02**	5/6	10
AgD2	A*29:02, **A*33:01, B*14:02, B*44:03, C*08:02, C*16:01**	5/6	10
AgD3	**A*30:02,A*68:02, B*41:01,** B*50:01, C*06:02, **C*17:01**	4/6	6
		**Average screened HLAs/donor: 4.66/6**	**Average screened clonotypes/donor: 8.66**
Ri01	**A*01:01, A*03:01, B*08:01, B*44:03, C*07:01, C*16:01**	6/6	4
Ri02	**A*02:01, A*26:01, B*18:01, B*51:01, C*07:01, C*14:02**	6/6	1
Ri03	**A*24:02,** A*31:01, **B*08:01, B*15:01, C*03:03, C*07:01**	5/6	3
Ri04	**A*01:01, A*24:02, B*08:01, B*35:01,** C*01:02, **C*07:01**	5/6	6
Ri05	**A*26:01, A*33:03,** B*38:01**, B*53:01, C*04:01, C*12:03**	5/6	2
Ri06	**A*03:01, A*24:02, B*08:01, B*51:01,** C*01:02, **C*07:01**	5/6	5
Ri07	**A*24:02 (homozygous), B*07:02, B*51:01, C*07:02, C*15:02**	6/6	1
		**Average screened HLAs/donor: 5.42/6**	**Average screened clonotypes/donor: 3.14**

[1]Assessed HLAs are underlined and in bold

[2]Clonotypes to test were identified based on scRNAseq analysis. All clones from cluster 1 with a) >10 $CD8^+$ T cells per clone, b) >60% of cells from this clone in cluster 1 were selected for testing

Supplementary Table 6: Summary of assessed HLA and tested clones from Figure 4d

Category	Specification	Manufacturer	Catalog#	Final concentration
PBMC isolation	Ficoll® Paque Plus	Sigma-Aldrich®	GE17-1440-02	--
	Fetal Bovine Serum (FBS)	Biowest	S1810	--
	Dimethyl sulfoxide (DMSO)	Sigma-Aldrich®	D2650	1:10
Cell culture	DMEM/F-12, GlutaMAX™ Supplement	Gibco™, Thermo Fisher Scientific	31331028	--
	B-27™ Supplement (50X), minus vitamin A	Gibco™, Thermo Fisher Scientific	12587001	1x
	N-2 Supplement (100X)	Gibco™, Thermo Fisher Scientific	17502001	1x
	Human EGF, premium grade	Miltenyi Biotec	130-097-750	10ng/ml
	Recombinant Human FGF-basic (154 a.a.)	Peprotech®	100-18B	10ng/ml
	Puromycin (solution)	InvivoGen	ant-pr-1	2µg/ml
	Doxycycline hyclate	Sigma-Aldrich®	D9891	2µg/ml
	DMEM, high glucose	Gibco™, Thermo Fisher Scientific	41965-039	--
	Cultrex Stem Cell Qualified Reduced Growth Factor Basement Membrane Extract	R&D Systems	3434-010-02	1:100
	Poly-L-ornithine solution	Sigma-Aldrich®	P4957	1:5
	Laminin from Engelbreth-Holm-Swarm murine sarcoma basement membrane	Sigma-Aldrich®	L2020	1:500
	TrypLE™ Express Enzyme (1X), phenol red	Gibco™, Thermo Fisher Scientific	12-605-010	--
Neuron chracterization	Paraformaldehyde 16% Aqueous Solution EM Grade	Electron Microscopy Sciences	15710	4:100
	Normal goat serum	Jackson ImmunoResearch	005-000-121	5:100
	Triton X-100	Sigma-Aldrich®	T9284	1:500
Neuron preparation	Human IFN-γ1b	Miltenyi Biotec	130-096-484	1ng/ml
	Recombinant Human TNF-alpha	R&D Systems	210-TA	1ng/ml
	EBV CD8^{+} T cell peptide pool (see Supplementary Table 4)	JPT Peptide Technologies	--	1µg/ml
	CMV CD8^{+} T cell peptide pool (see Supplementary Table 4)	JPT Peptide Technologies	--	1µg/ml
	PepMix™ VZV (IE63)	JPT Peptide Technologies	PM-VZV-IE63	1µg/ml
PBMC/CD8^{+} T cell cultures with neurons	CTS™ OpTmizer™ T Cell Expansion SFM	Gibco™, Thermo Fisher Scientific	A1048501	--
	Human IL-2 IS, premium grade	Miltenyi Biotec	130-097-746	1000UI/ml

Flow cytometry	Fetal Bovine Serum (FBS)	Biowest	S1810	2:100
	Paraformaldehyde 16% Aqueous Solution EM Grade	Electron Microscopy Sciences	15710	4:100
MACS & IFN-γ secretion assay	Bovine Serum Albumin (BSA)	Sigma-Aldrich®	A3912	1:200
TCR repertoire sequencing	Lysis/Binding Buffer	Invitrogen™, Thermo Fisher Scientific	A33562	--
Single-cell TCR sequencing	UltraPure™ DNase/RNase-Free Distilled Water	Invitrogen™, Thermo Fisher Scientific	10977-035	--
	Triton X-100	Sigma-Aldrich®	T9284	1:500
	RNAse Inhibitor, 40'000 U/ml	Enymatics	Y9240L	2U/ul
	One-Step RT-PCR kit	QIAGEN	210212	--
	AMPure XP Bead-Based Reagent	Beckman Coulter	A63881	--
	Phusion® Hot Start Flex DNA Polymerase	New England Biolabs®	M0535S	--
	dNTP Mix	Promega	U1511	--
	Affymetrix™ ExoSAP-IT™ For PCR Product Clean-Up	Fisher Scientific, Thermo Fisher Scientific	15513687	--
TCR validation	HiScribe® T7 ARCA mRNA Kit (with tailing)	New England Biolabs®	E2060S	--
	Neon Transfection System 10 µl	Thermo Fisher Scientific	MPK1096	--
	Bio-Glo Luciferase Assay System	Promega	G7940	--

PBMC: peripheral blood mononuclear cells,
MACS: Magnetic-associated cell sorting

Supplementary Table 7: Media, reagents, cytokines and peptides used in this study

Category	Specification	Target	Clone	Conjugate/Color	Manufacturer	Catalog#	Final concentration
Neuron characterization	Anti-Neurofilament 200 antibody produced in rabbit	NF-200	Polyclonal	unconjugated	Sigma-Aldrich®	N4142	1:200
	Anti-MAP2 antibody	MAP2	Polyclonal	unconjugated	Abcam	ab5392	1:200
	Mouse monoclonal antibody to Human MHC Class I (HLA-A, HLA-B, HLA-C)	HLA-A, -B, -C	W6/32	unconjugated	AffinityImmuno	ab-131-060	1:500
	4′,6-Diamidine-2′-phenylindole dihydrochloride (DAPI)	dsDNA	--	--	Sigma-Aldrich®, Roche	10236276001	1:500
	Donkey anti-Rabbit IgG (H+L) Secondary Antibody, Alexa Fluor™ 546	IgG	Polyclonal	AF546	Invitrogen™, Thermo Fisher Scientific	A10040	1:200
	Goat anti-Chicken IgY (H+L) Secondary Antibody, Alexa Fluor™ 546	IgY	Polyclonal	AF546	Invitrogen™, Thermo Fisher Scientific	A11040	1:200
	Donkey anti-Mouse IgG (H+L) Secondary Antibody, Alexa Fluor™ 488	IgG	Polyclonal	AF488	Invitrogen™, Thermo Fisher Scientific	A21202	1:200
Magnetic-associated cell sorting (MACS)	$CD8^+$ T cell Biotin-Antibody Cocktail human	cf. Manufacturer details	cf. Manufacturer details	unconjugated	Miltenyi Biotec	130-096-495	10µl/10mio cells
	$CD8^+$ T cell Microbead cocktail human	cf. Manufacturer details	cf. Manufacturer details	Magnetic Microbeads	Miltenyi Biotec	130-096-495	20µl/10mio cells
IFN-γ secretion assay	IFN-γ Catch Reagent, human	CD45, IFN-γ	Unspecified	unconjugated	Miltenyi Biotec	130-090-433	1:10
	IFN-γ Detection Antibody, human	IFN-γ	Unspecified	FITC	Miltenyi Biotec	130-090-433	10µl/ml
Flow cytometry	MHC class I Antibody	HLA-A, -B, -C	W6/32	FITC	Santa Cruz Biotechnology	sc-32235	2:100
	Mouse Anti-Human CD8	CD8α	RPA-T8	Pacific Blue	BD Biosciences	558207	2:100
	Mouse Anti-Human CD3	CD3	SK7	APC-H7	BD Biosciences	560176	2:100

	LIVE/DEAD™ Fixable Aqua Dead Cell Stain Kit	Free amines	--	Aqua	Invitrogen™, Thermo Fisher Scientific	L34957	4:1000
	NA/LE Mouse Anti-Human CD28	CD28	CD28.2	unconjugated	BD Biosciences	555725	5µg/ml
TCR validation	Mouse monoclonal antibody to Human MHC Class I (HLA-A, HLA-B, HLA-C)	HLA-A, -B, -C	W6/32	unconjugated	AffinityImmuno	ab-131-060	50µg/ml
	HLA-BC Monoclonal Antibody, functional grade	HLA-B, -C	B1.23.2	unconjugated	Invitrogen™, Thermo Fisher Scientific	16-5935-82	50µg/ml
	T Cell TransAct™ human	CD3, CD28	Unspecified	unconjugated	Miltenyi Biotec	130-111-160	1:100
Histology	Anti-CD8 alpha antibody	CD8α	C8-144B	unconjugated	Abcam	ab17147	1:50
	TOX Monoclonal Antibody (TXRX10), eBioscience™	TOX	TXRX10	unconjugated	Invitrogen™, Thermo Fisher Scientific	14-6502-82	--
	Goat anti-Mouse IgG1 Secondary Antibody, Alexa Fluor™ 647	IgG1	Polyclonal	AF647	Invitrogen™, Thermo Fisher Scientific	A21240	--
	TSA Vivid™ Fluorophore Kit 570	--	--	--	Bio-Techne	7526	1:300

Supplementary Table 8: Antibodies and dyes used in this study
