# Supplementary material for: Neuron-reactive KIR+CD8+ T cells display an encephalitogenic transcriptional program in autoimmune encephalitis
Source: Nat Commun. 2025 Sep 29;16:8568. doi: 10.1038/s41467-025-63573-1 (PMC12479921; doi:10.1038/s41467-025-63573-1)
Supplement: Supplementary file 2 — Description of Additional Supplementary Files [file 41467_2025_63573_MOESM2_ESM.pdf]

Description of Additional Supplementary Files

Supplementary Data 1. List of all cloned and tested TCRs used in Figure 4

Supplementary Data 2. Differential gene expression analysis of all clusters performed on single cell data

Supplementary Data 3. Differential gene expression analysis of neuron-reactive CD8+ T cells from Ri AIE vs AgD performed on single cell data
